# Multimodal ultrasound deep learning to detect fibrosis in early chronic kidney disease

**DOI:** 10.1080/0886022X.2024.2417740

**Published:** 2024-10-22

**Authors:** Xiachuan Qin, Xiaoling Liu, Linlin Xia, Qi Luo, Chaoxue Zhang

**Affiliations:** a Department of Ultrasound, Chengdu Second People’s Hospital, Chengdu, Sichuan Province, China; b Department of Ultrasound, Nanchong Central Hospital, The Second Clinical Medical College, North Sichuan Medical College (University), Nanchong, Sichuan Province, China; c Department of Ultrasound, The First Affiliated Hospital of Anhui Medical University, Hefei, Anhui Province, China

**Keywords:** Chronic kidney disease, deep learning, fibrosis, multimodal, ultrasound

## Abstract

We developed a multimodal ultrasound (US) deep learning (DL) fusion model to automatically classify early fibrosis in patients with chronic kidney disease (CKD). This prospective study included patients with CKD who underwent continuous gray-scale US, superb microvascular imaging, and strain elastography from May to November 2022. According to the pathological tubular atrophy and interstitial fibrosis score, patients were divided into minimal and mild groups (affected area ≤10% and 11 − 25% of the total cortical volume, respectively). The dataset was divided into training (70%) and test (30%) sets. A DL model combining the features of the three US modes was developed to predict early fibrosis in patients with CKD. We compared these findings with the area under the receiver operating characteristic curve (AUC) of the clinical model by analyzing the receiver operating characteristic curve in the test set. The AUC of single-mode DL based on gray-scale US, superb microvascular imaging, and strain elastography was 0.682, 0.745, and 0.648, respectively, while that of the multimodal US DL model was 0.86. The accuracy, specificity, and sensitivity of the multimodal US DL model were 0.779, 0.767, and 0.796, respectively, and the negative and positive predictive values were 0.842 and 0.706, respectively. The AUC of the multimodal US DL model was significantly better than that of the single-mode DL and clinical models. The DL algorithm developed using multimodal US images can effectively predict early fibrosis in patients with CKD with significantly greater accuracy than single-mode DL or clinical models.

## Introduction

Renal fibrosis is an important histopathological feature of chronic kidney disease (CKD), closely related to the deterioration of renal function [1,2]. The severity of renal fibrosis is the most important clinical risk factor for poor prognosis in patients with CKD [2,3] and is the best predictor of CKD progression [4]. Renal biopsy is the gold standard for evaluating the degree of renal fibrosis. However, this invasive technique may lead to complications such as bleeding and even death in a few patients [5,6] and is unsuitable for long-term monitoring of disease progression [7]. Therefore, current research has been investigating the degree of renal fibrosis through noninvasive imaging methods, with satisfactory results [8–10]. However, all studies to date have focused on the difference between mild and moderate/severe renal fibrosis (0 − 25% vs. 25%), often ignoring the minimal degree of pathological grading (≤10%) [11,12]. Accurate detection of early renal fibrosis is advantageous for detecting early disease and allows appropriate treatment to begin sooner to prevent disease progression [11,13]. However, distinguishing between minimal and mild fibrosis is challenging.

Renal ultrasound(US) is generally considered the first-line imaging technique for evaluating the degree of CKD^6^. Compared with other imaging methods (such as radiography, computed tomography, and magnetic resonance imaging), US is safer, more cost-effective, avoids radiation, and can be viewed in real time. Chen et al. thought that the elasto­graphy value would be a valuable biomarker to assess moderate-to-severe renal impairment [14]. Superb micro­vascular imaging (SMI) uses an adaptive wall filter different from color Dopper imaging and minimizes flash artifacts. Even without the use of any contrast agent, SMI can improve slow flow visibility and sensitivity of small vessel signal detection [15,16]. SMI can display small blood vessels better than other Doppler techniques, assessing the degree of fibrosis by evaluating peripheral vascular thinning, increased tortuosity, and vascular tree passivation [17,18].

Although current research suggests that the length, echo, blood flow, and hardness of the kidney are related to the degree of renal fibrosis, these characteristics are still not effective in ascertaining renal fibrosis [18–22]. Recently, deep learning (DL) has been increasingly applied in image-based medical diagnoses [9,23,24]. It can learn the most representative features directly from the original image pixels, has a more complex and easily expandable structure, does not need to define intermediate steps manually [25], and can build models that better represent lesions than radiologists [26,27]. Renal changes are not obvious, especially in the early stages. A single modality can reflect limited information. Multimodal studies based on DL can utilize richer information encoded in multimodal data, achieving better results than single-mode DL [28,29].

In this study, we prospectively collected gray-scale US, SMI, and strain elastography (SE) continuous scans of the kidneys of patients with CKD. We aimed to develop a multimodal US DL fusion model for the automatic classification of early fibrosis in patients with CKD and to compare it with single-mode US DL and clinical prediction models.

## Methods

This prospective study was conducted in accordance with the Declaration of Helsinki and was approved by the Internal Review Committee of our institution. Written informed consent was obtained from all participants. From May to November 2022, we recruited 114 patients with CKD confirmed by renal biopsy (average age, 48; range 24 − 81 years) from the Hospital. The inclusion criteria were as follows: (1) at least 10 glomeruli in the specimen visible under a light microscope; (2) aged >18 years; and (3) distance between the kidney and skin of ≤5 cm.

The exclusion criteria were as follows: (1) presence of acute renal damage, heart valve disease, or heart failure; (2) urinary tract obstruction; (3) kidney cysts or tumors; (4) Doppler patterns suggestive of renal artery stenosis [30]; and (5) patients who could not cooperate with breath hold instructions.

**Figure 1.**
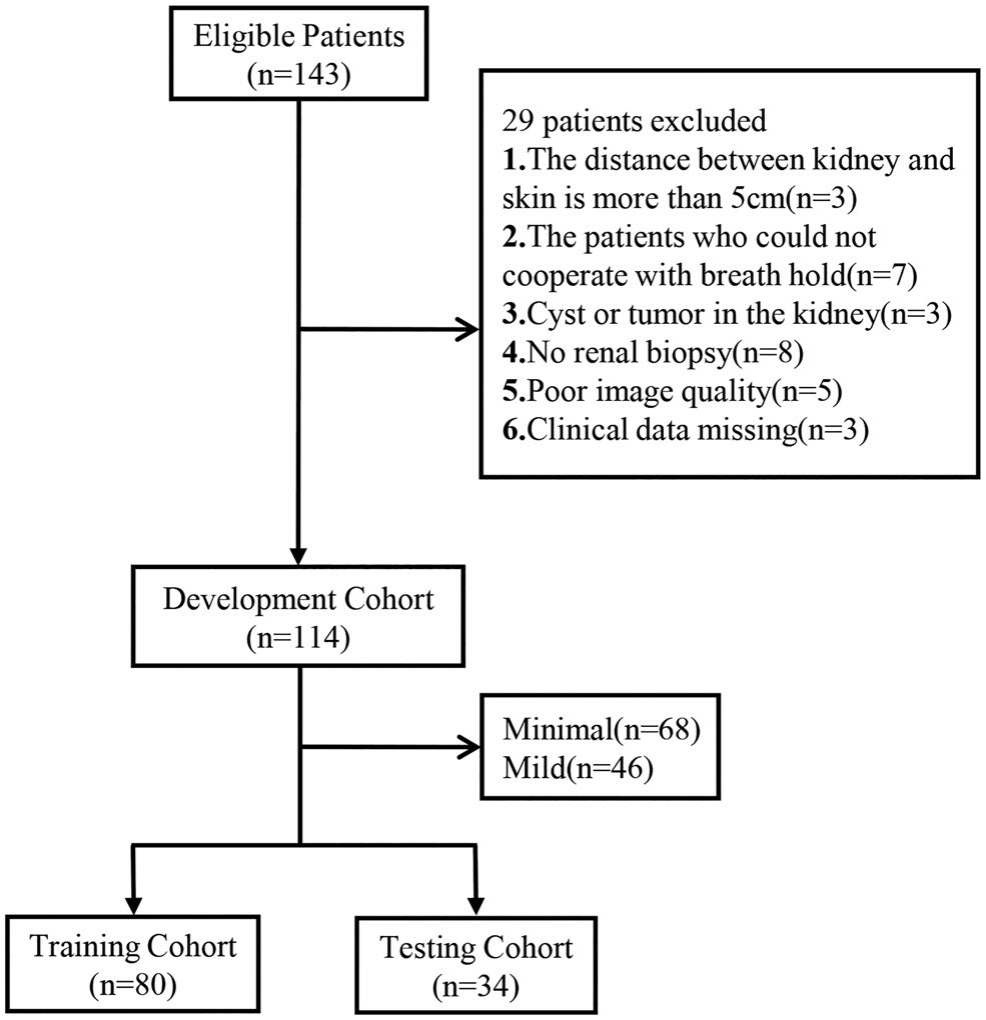
Patient flowchart for this study. TA/IF: tubular pathology/interstitial fibrosis.

The study flow diagram is shown in Figure 1. Participants in the development dataset were randomly assigned to a training (70%) or testing (30%) cohort.

### US examination

All patients underwent both US examination and biopsy of the right kidney. An experienced doctor (with 16 years of experience in renal US examination) collected the images one day before the patient underwent a renal biopsy. An Aplio i700 Canon US system (Canon Medical Systems, Otawara, Japan) with a 3.5 MHz curvilinear transducer (i8C1) was utilized. Appendix A describes the image acquisition process. For all cases, gray-scale US, SMI, and SE continuous images in the largest longitudinal direction of the kidney were obtained. A complete description of the multimodal US image collection is provided in Appendix A.

### DL analysis

Before the image was introduced into the DL network, two experienced doctors (with 24 years and 8 of experience in performing renal US, respectively) performed a segmentation mask for all images. It include the entire renal parenchyma. Additionally, we adjusted the size of the segmentation mask to the maximum to maintain sufficient edges. Through segmentation, we manually eliminated irrelevant information, such as text and instrument settings, and ensured similar lesion and mask proportions in each image. Finally, all the captured US images were adjusted to an aspect ratio of 224 × 224 for image quality control and network input.

**Figure 2.**
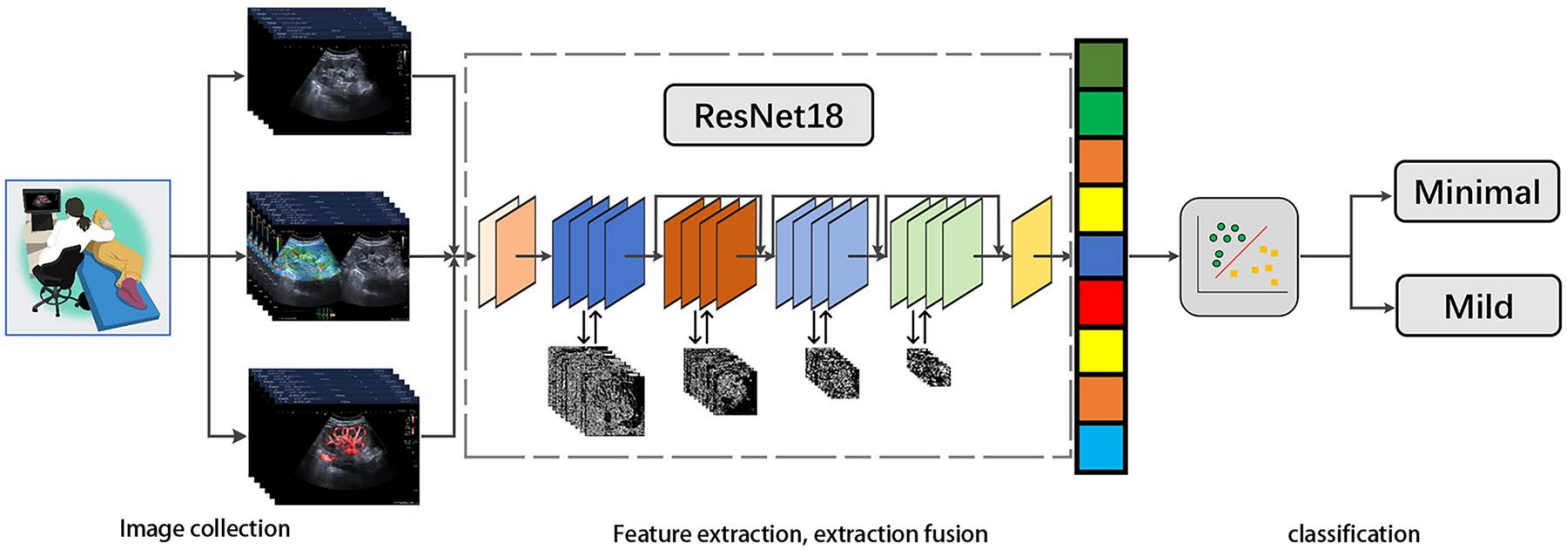
Flowchart of procedures in the development and evaluation of DL model for automated early fibrosis prediction.

We used ResNet18 as the network model [31], and the US, SMI, and SE images were used as the input of the network model. We used the classification methods of single-mode and multimodal fusion. In single-mode classification, we directly trained the kidney disease recognition network using data from each imaging mode separately. The multimodal classification used a early fusion strategy. The images of the three modes were spliced to create the multimodal network model input, which enabled the kidney disease recognition network to extract multimodal information related to kidney disease. This reduced the limitations of single-mode disease recognition networks, which have poor final recognition accuracy. The convolutional neural network architecture is shown in Figure 2.

Owing to the small dataset available, we used migration learning and fine-tuning to better classify the dataset. Using the pre-trained model for feature extraction, we used the weight obtained from training on the ImageNet dataset as the pre-training model. In the early stage of the convolution neural network development, the features extracted by the model were similar, generally global information such as edges and textures. Therefore, using the pre-training model can accelerate convergence and improve the generalizability of the network model. A complete description of the image preprocessing and deep-learning algorithms is provided in Appendix E1.

In order to overcome the ‘black box’ property of DL models, the SHAP method is adopted to explain the contribution value of each modality to the DL model [32]. This study uses a pie chart to represent the global interpretation of the model, which displays the average SHAP values of three different input modalities.

### Pathological image analysis

The renal biopsy samples of the participants were assessed under a light microscope by two experienced renal pathologists (with 6 and 20 years of experience, respectively) who provided semi-quantitative scores for histopathological lesions. The system provides a classification score based on the percentage of the cortex area with interstitial fibrosis and tubular atrophy (IF/TA) (0 − 100%) and then converts it to a semi-quantitative grade according to the IF/TA score. The IF/TA score was divided into two groups: minimal (affected area accounts for ≤10% of the total cortical volume) and mild (affected area accounts for 11 − 25% of the total cortical volume) [11,33].

### Clinical prediction model

The eGFR and 24-h proteinuria are used to assess kidney function. The change in the eGFR and 24-h proteinuria were related to the development of kidney cortical IF/TA [34,35]. Therefore, we used logistic regression algorithm to integrate and estimate the key indicators of eGFR and 24-h urinary protein. The clinical model used the weighted values of these two parameters to predict early renal fibrosis and compared it with the DL model.

### Statistical analyses

All statistical analyses were conducted using SPSS 26.0 (IBM Corp., Armonk, NY, USA) and Python 2.7 (Python Software Foundation, Beaverton, OR, USA). Quantitative data adhering to a normal distribution was expressed as mean ± standard deviation and assessed using independent samples t-test; non-normally distributed quantitative data was depicted as median ± interquartile range and evaluated using the Mann-Whitney U test. Categorical variables were presented in terms of numbers and percentages and subjected to the chi-square test or Fisher’s exact test, as appropriate. Statistical significance was set at *p* < 0.05.To evaluate and compare the performance of the DL models and the clinical model, we constructed receiver operating characteristic (ROC) curves and calculated the area under the ROC curve (AUC) for each. The AUC values were further compared using DeLong’s test to ascertain the relative performance of the models. The optimal cutoff value derived from the ROC curve was applied to the test set to determine the accuracy, specificity, sensitivity, and both negative and positive predictive values(NPV and PPV) for the detection of early fibrosis. To evaluate the clinical effectiveness of the models, decision curve analysis (DCA) was performed by calculating the net benefit in different probability threshold of the testing cohorts.

## Results

### Demographic baseline and clinical model

Fifty-two men and 62 women (mean age: 41.2 ± 13.4 years) with CKD underwent renal biopsy, and 68/114 (59.6%) had an IF/TA score of ≤10%, while 46/114 (40.4%) had an IF/TA score of 11–25%. After random distribution, the patients were divided into a training queue (2922 pictures of 80 patients, including 974 US images, 974 SMI images, and 974 SE images) and a test queue (1140 pictures of 34 patients, including 380 US images, 380 SMI images, and 380 SE images). In the training queue, 48/80 (60%) patients had an IF/TA score of ≤10%, and 32/80 (40%) had an IF/TA score of 11 − 25%. In the test queue, 20/34 (58.8%) patients had an IF/TA score of ≤10%, and 14/34 (41.2%) had an IF/TA score of 11 − 25%. Patient and baseline characteristics are shown in Table 1.

**Table 1.** Clinical factors in the training and testing cohorts.

	Training cohort (*n* = 80)	Testing cohort (*n* = 34)	P
Age(years)	40.8 ± 12.6	42.2 ± 15.2	0.504
Sex(male/female)	34/46	18/16	0.179
TA/IF(minimal/mild)	48/32	20/14	0.973
Renal length(mm)	106.9 ± 8.6	105.1 ± 8.8	0.806
Cortex echo(hyper/hypo)	55/25	17/17	0.123
PSV of MRA(cm/s)	74.2 ± 16.1	75.8 ± 20.7	0.529
RI of MRA	0.67 ± 0.06	0.66 ± 0.06	0.742
Mean arterial pressure(mmHg)	101.1 ± 11.8	100 ± 12.6	0.413
eGFR at biopsy(mL/min/1.73m^2^)	93.6 ± 28.9	99.4 ± 27.5	0.257
Creatinine at biopsy(umol/L)	83.6 ± 25.9	80 ± 25	0.315
24h Urine protein at biopsy(g/24h)	2.6 ± 3.7	3.7 ± 4	0.252
Uric acid at biopsy(umol/L)	370.1 ± 100.5	390.4 ± 114.9	0.475

TA/IF: Tubular pathology/interstitial fibrosis; MRA: main renal artery; RI: resistance index; PSV: peak systolic velocity.

### Single-mode DL model performance

The DL model based on SMI images had the best results, with an AUC of 0.745 and an accuracy of 0.671. The AUC based on US images of the kidney was 0.682, and the accuracy was 0.65. The AUC of SE images was 0.648, and the accuracy was 0.621 (Table 2).

**Table 2.** Performance of four DL models and clinical model in the testing cohort.

Different models	Multimodal DL model	US DL model	SE DL model	SMI DL model	Clinical model
AUC	0.86	0.682	0.648	0.745	0.800
ACC	77.9	65	62.1	67.1	71.0
SEN	79.6	59.9	57.3	87.3	81.8
SPE	76.7	68.6	65.5	52.9	65.0
NPV	84.2	70.8	68.5	85.5	86.7
PPV	70.6	57.3	53.9	56.6	56.2
F1 score	74.8	58.6	56	68.7	66.7
Delong *P*-value	/	<0.001	<0.001	<0.001	0.473

DL: deep learning; US: ultrasound; SE: strain elasticity; SMI: superb microvascular imaging; AUC: area under the curve; ACC: accuracy; SEN: sensitivity; SPE: specificity; NPV: negative predictive value; PPV: positive predictive value.

Delong *P*-value is compared with Multimodal DL model in the testing cohort.

### Comparison of multimodal DL model performance and eGFR clinical model

The AUC of the multimodal DL was 0.86.The accuracy, specificity, and sensitivity were, 0.779, 0.767, and 0.796, respectively. The NPV and PPV were 0.843 and 0.706, respectively.

**Figure 3.**
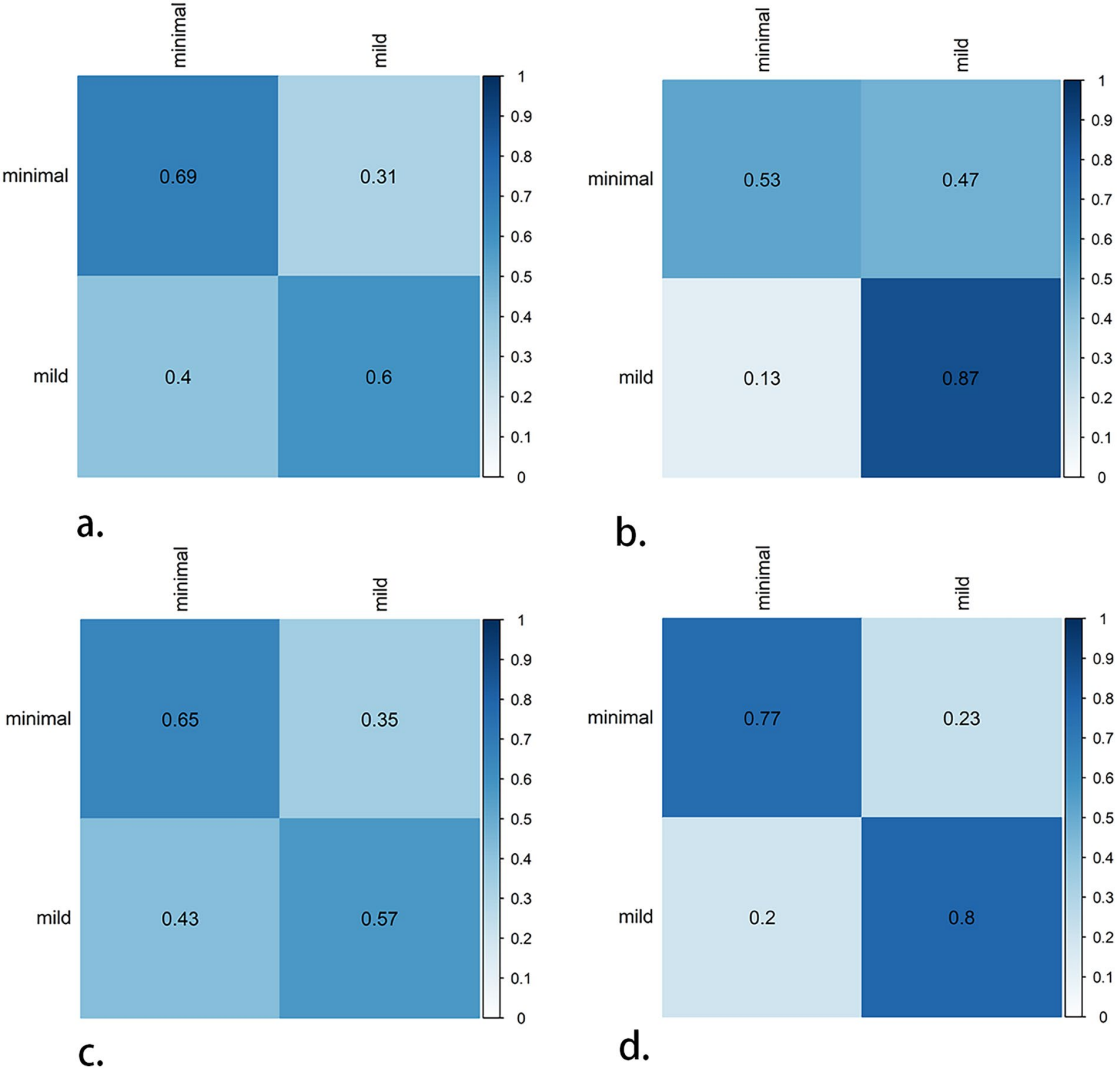
Confusion matrices of the diagnostic performance in the gray-scale DL model(a), superb microvascular imaging DL model (b), strain elasticity DL model (c) and multimodal DL model(d). The true label as diagnosed by using histopathologic analysis is used as the reference standard. The predicted label as given by the DL model is also shown. The bars on the right represent the relative frequency in the test set, and the corresponding color coding is shown within the matrices.

Figure 3 shows the confusion matrix of all DL models used to distinguish the minimal and mild groups in the test queue. Notably, the multimodal DL model diagnosis of minimal disease (0.77) was higher than that of the US (0.69), SE (0.53), or SMI model (0.65). The multimodal DL model has statistical significance compared to the other three unimodal models(*p* < 0.05)

**Figure 4.**
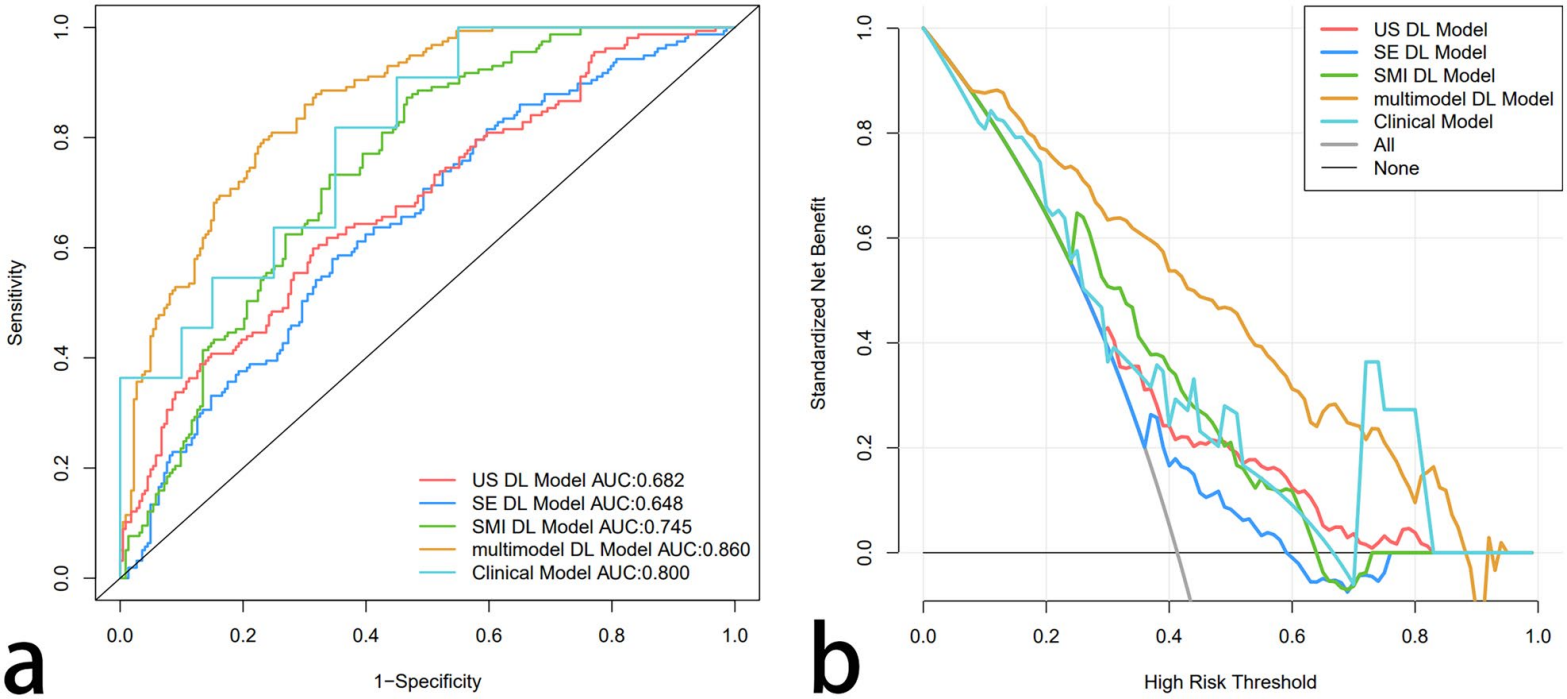
ROC Curves and DCA curves of the five models. (a) The ROC curves of the five models. (b) The DCA curves of the five models.

As shown in Table 2 and Figure 4, in the test group, the AUC of the clinical model for predicting early chronic renal fibrosis was 0.800, the accuracy was 0.71, the specificity was 0.65, and the sensitivity was 0.82; the NPV and PPV were 0.867 and 0.562, respectively. Although there is no statistical significance between multimodal DL model and clinical model(*p* = 0.47), we can find that multimodal DL has better performance than clinical model. The DCA confirms the fusion DL model provides more clinical benefit than the clinical model (Figure 3).

### Interpretability of DL model

**Figure 5.**
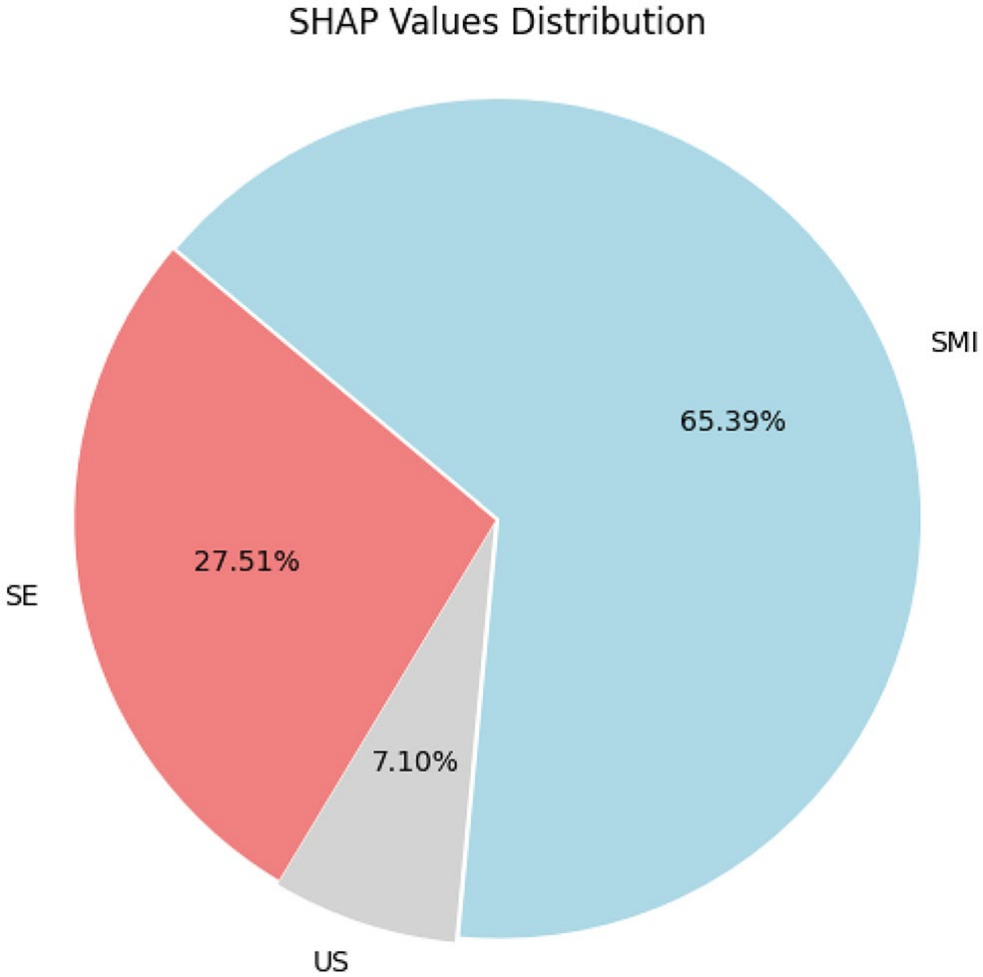
In the global SHAP interpretation, it can be noted that the SMI accounts for 65.4%, the SE accounting for 27.5%, and the US accounting for only 7.1%.

This study uses a global SHAP interpretation model to reveal the importance and impact of each modality on the final diagnosis of the optimal model. Through the global SHAP pie chart display, it can be seen that the sector area representing SMI occupies the main proportion in the entire circle, accounting for 65.4%; Next is SE, which accounts for 27.5% of the overall area; The US has the lowest contribution in the entire decision-making process, accounting for only 7.1% (Figure 5).

## Discussion

In this study, we established a fusion DL model based on multimodal US to predict the degree of fibrosis in the early stages of CKD. The model produced satisfactory prediction results: the AUC of the test group was 0.86, and the accuracy was 0.779. The ROC performance of this model was better than that of the single-mode DL and clinical prediction models. Our results demonstrate the feasibility of using a multimodal DL model to predict mild IF/TA in patients with CKD. To the best of our knowledge, this is the first study to use the fusion DL model of multimodal US to predict early fibrosis in patients with CKD.

Renal fibrosis mainly refers to interstitial renal fibrosis and is often accompanied by vascular occlusion, glomerulosclerosis, renal tubular atrophy, and arteriosclerosis [13]. In the early stage of renal IF, the permeability of interstitial capillaries increases, and the gradual decrease in the surface area of interstitial capillaries leads to hypoxia in the kidney and affects cell function [6]. Conventional US can reflect the size, echo, and cortical thickness of the kidneys, but the information it can provide is often limited. Therefore, we included SMI and SE imaging modes in the model. SMI is an advanced Doppler US technique [18,36,37]. Zaher Armaly et al. collected 6 CKD patients who underwent puncture biopsy for SMI examination and found that patients with higher levels of IF had lower SMI vascular index [18]. SMI represents the microvascular situation of the kidney.SE was used to evaluate tissue hardness. It repeatedly exerts a slight pressure on the target tissue. It quantifies tissue elasticity by measuring deformation. Jing Gao et al. used SE to evaluate the degree of IF in kidney transplant patients, and the elastic strain value of the renal cortex was closely related to the degree of IF in the renal cortex [38].The qualitative color imaging obtained by SE is easy to perform, and the images are easy to analyze [39–41], SE represents the hardness of the kidney. The biological characteristics of renal IF are complex and interact with many factors. Multimodal data fusion can extract and combine multi-scale information from different modalities. Owing to difficulty obtaining clinically relevant results with the single-mode model, we attempted to integrate the three modes to predict early fibrosis in patients with CKD.

DL can be used to decode the quantitative features of digital US images. This system automatically focuses on the basic features, recognizing features that cannot be detected by human eyes, and conducts independent diagnoses through convolution neural networks [26,42]. Two recent studies using DL to predict renal fibrosis were conducted on a single-mode US image [9,10]. First, we established single-mode US DL models using US, SMI, or SE to identify early renal fibrosis. SMI obtained the best ROC results for predicting early renal fibrosis, although the results were still not clinically relevant. A multimodal method is considered to be better than a single-mode method [43,44]. Therefore, we fused the three modes to establish a multimodal DL model and integrated the characteristic information of all modes from the input level. Through an experimental comparison, we found that the multimodal kidney disease identification network achieved an accuracy of 0.779 in the kidney test set. The accuracy of the multimodal kidney recognition network increased by 0.129, 0.158, and 0.108, respectively, compared with the US, SE, and SMI single-mode models in the test set. The multimodal feature allows the convolution neural network to extract more useful features to achieve increased diagnostic accuracy and to improve the generalizability of the network model. For a single-mode input, the features extracted by the convolutional neural network have certain limitations, which are not conducive to improving the final recognition accuracy on the test set.

The eGFR and 24-h urinary protein represents the contribution of glomeruli to renal excretion and is the clearest characteristic index used to reflect the degree of CKD [34,35]. They are also an important predictor of renal fibrosis [45,46]. We established a clinical model using eGFR and 24-h urine protein data. In our study, the AUC of the test group in which the clinical model predicted early renal fibrosis was 0.80. However, our in-depth learning model based on multimodal US can achieve the results are superior to those of the clinical model. This further emphasizes the effectiveness of our in-depth learning model and its significant clinical application value in predicting early fibrosis.

Although DL models can make accurate diagnoses of lesions, they work like black boxes and the diagnostic process is unknown. But if the machine cannot explain how it makes judgments, it not only confuses doctors and patients, but also restricts its clinical application [47].In this study, we used a pie chart interpreted by global SHAP to measure the contribution of three US modalities in diagnosing early IF degree in CKD patients. We can see that SMI plays a major role in decision-making in the global SHAP value, accounting for 65.4%. The results also indicate that SMI, which represents renal blood flow perfusion, plays an important role in determining the IF degree of CKD. SE plays a 27.5% role in decision-making, which further confirms previous research that multiple factors in CKD jointly affect the elasticity of the kidneys. Blood perfusion is the main factor affecting elasticity, but the degree of IF is still involved [48]. Although the role of US in the model only accounts for 7.1% of the total proportion, we believe there are two reasons for this: 1. The patients selected for the study were those with minimal IF and mild IF, and the pathological changes caused by mild changes in kidney morphology were not significant, resulting in very limited diagnostic efficacy of US for it; 2. Due to the use of colored SMI mode in this study to replace some of the functions of US, the proportion of US has decreased. In short, through global SHAP, we can clearly understand the role of each modality in the diagnostic process, and this functional visualization method provides more confidence in the diagnostic ability of the model.

Our study had some limitations. First, this was a pilot study conducted at a single academic institution, which limits its generalizability. Second, for this proof-of-concept study, we focused only on the differences in early renal fibrosis (≤10% vs. 11 − 25%); the moderate (26–50%) and severe (>50%) groups were not included. In the future, a larger sample size is needed to quantitatively grade renal fibrosis.

The fusion DL model established by combining gray-scale US, SMI, and SE showed good accuracy in the noninvasive prediction of early chronic renal fibrosis. The performance of the multimodal model was significantly better than that of the monomodal and clinical prediction models. It can provide clinically useful information for individualized diagnosis and treatment of patients with early CKD. This should reduce the cost of additional kidney biopsies with significant clinical value in predicting early fibrosis.

## Supplementary Material

fig5_R1.tif

Supplemental file for Review.docx

fig3_R1.tif

fig4_R1.tif

Appendix A.docx

fig2_R1.tif

fig1_R1.tif
