# Supplementary figures and images for: Multimodal ultrasound deep learning to detect fibrosis in early chronic kidney disease
Source: Ren Fail. 2024 Oct 22;46(2):2417740. doi: 10.1080/0886022X.2024.2417740 (PMC11497579; doi:10.1080/0886022X.2024.2417740)

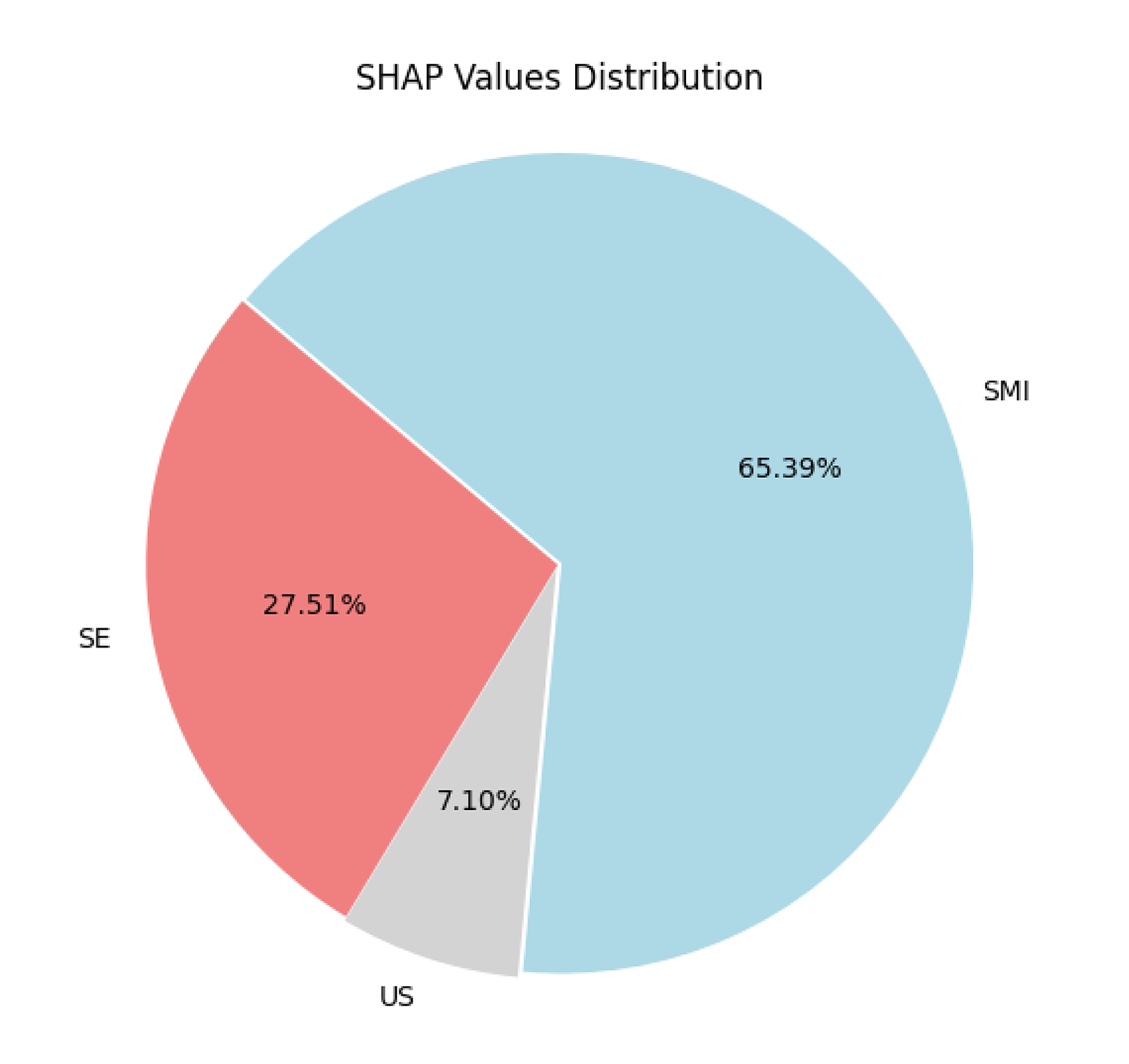

Supplement: fig5_R1.tif [file IRNF_A_2417740_SM4848.tif]

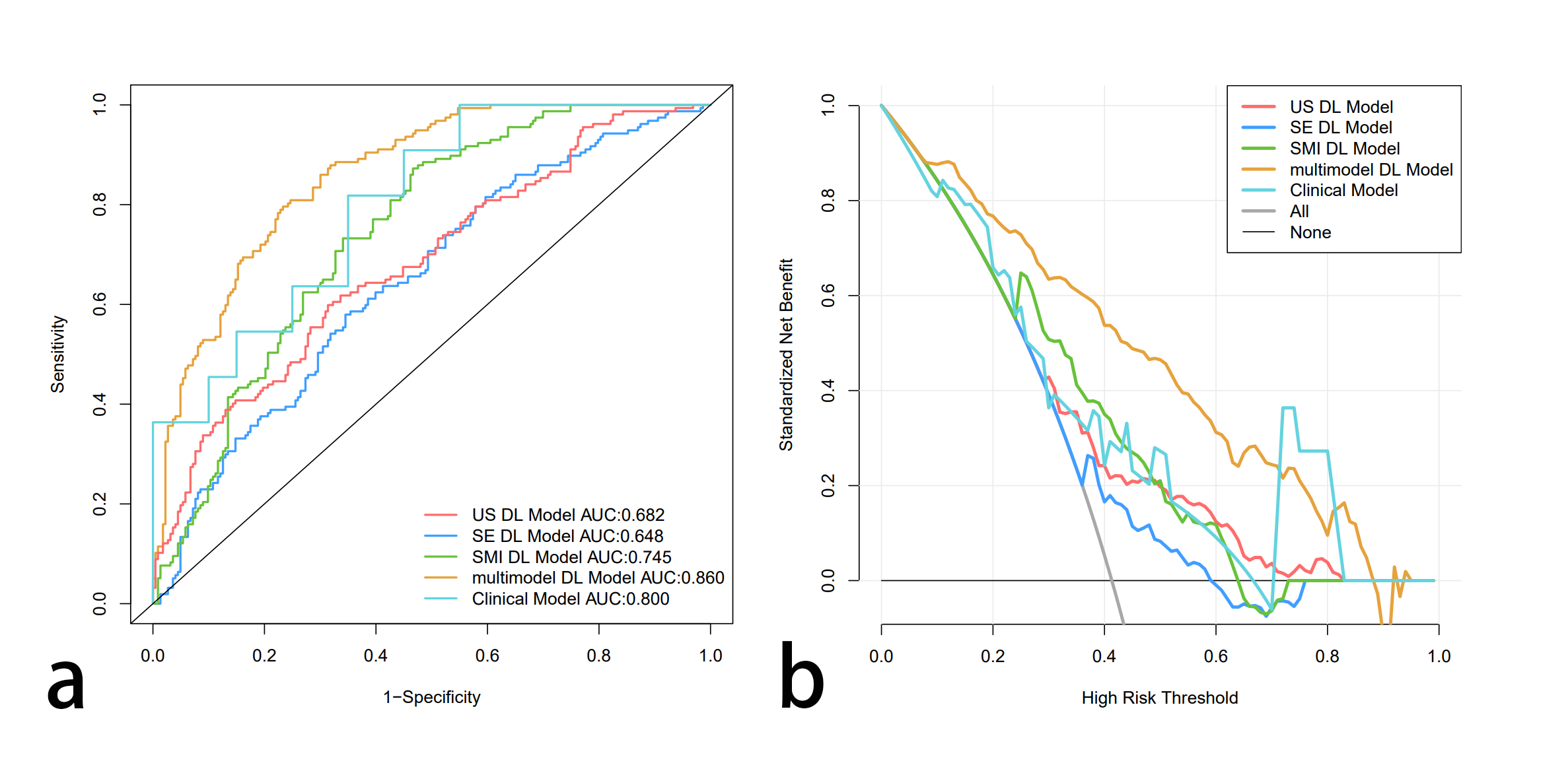

Supplement: fig3_R1.tif [file IRNF_A_2417740_SM4846.tif]

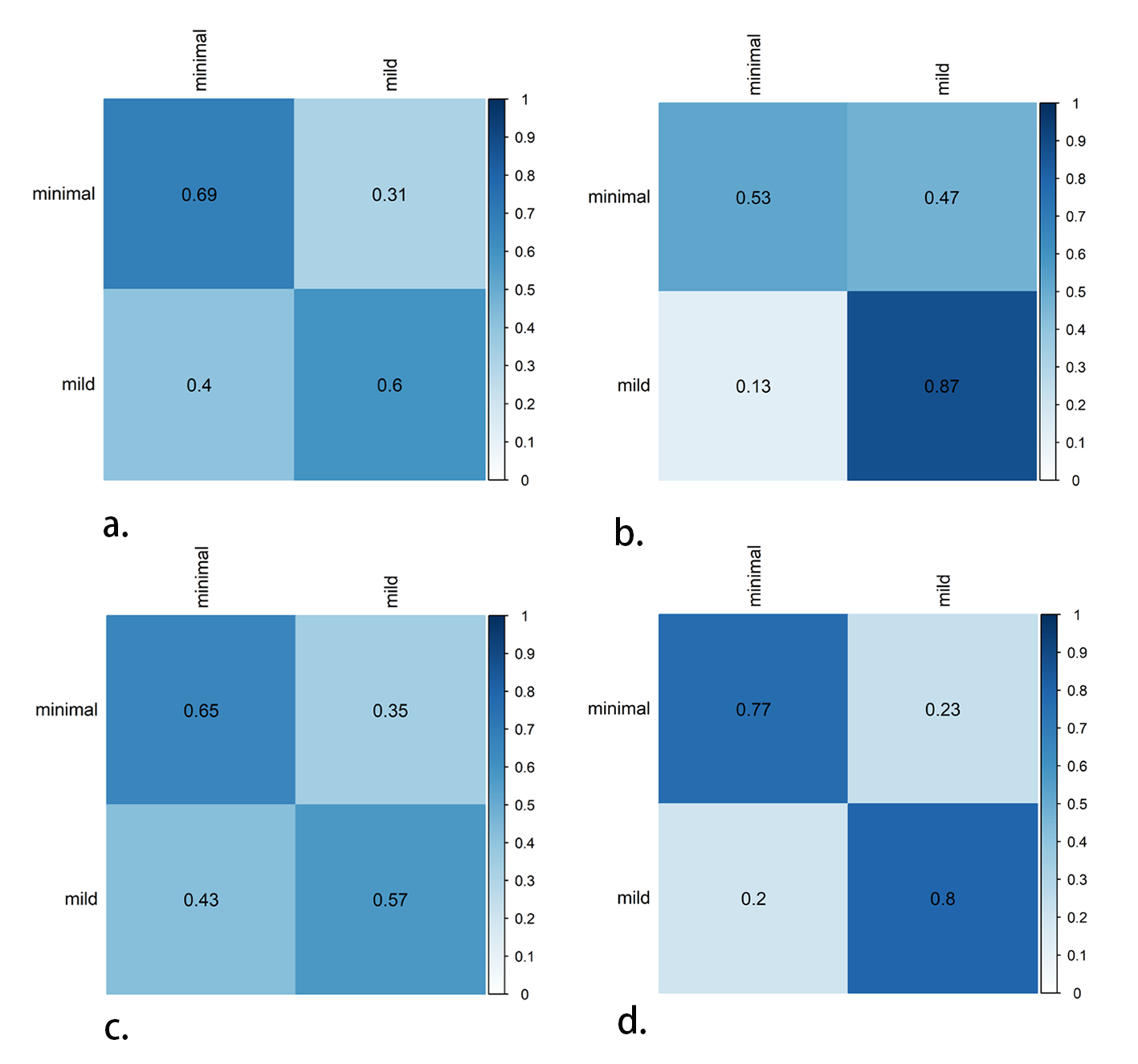

Supplement: fig4_R1.tif [file IRNF_A_2417740_SM4845.tif]

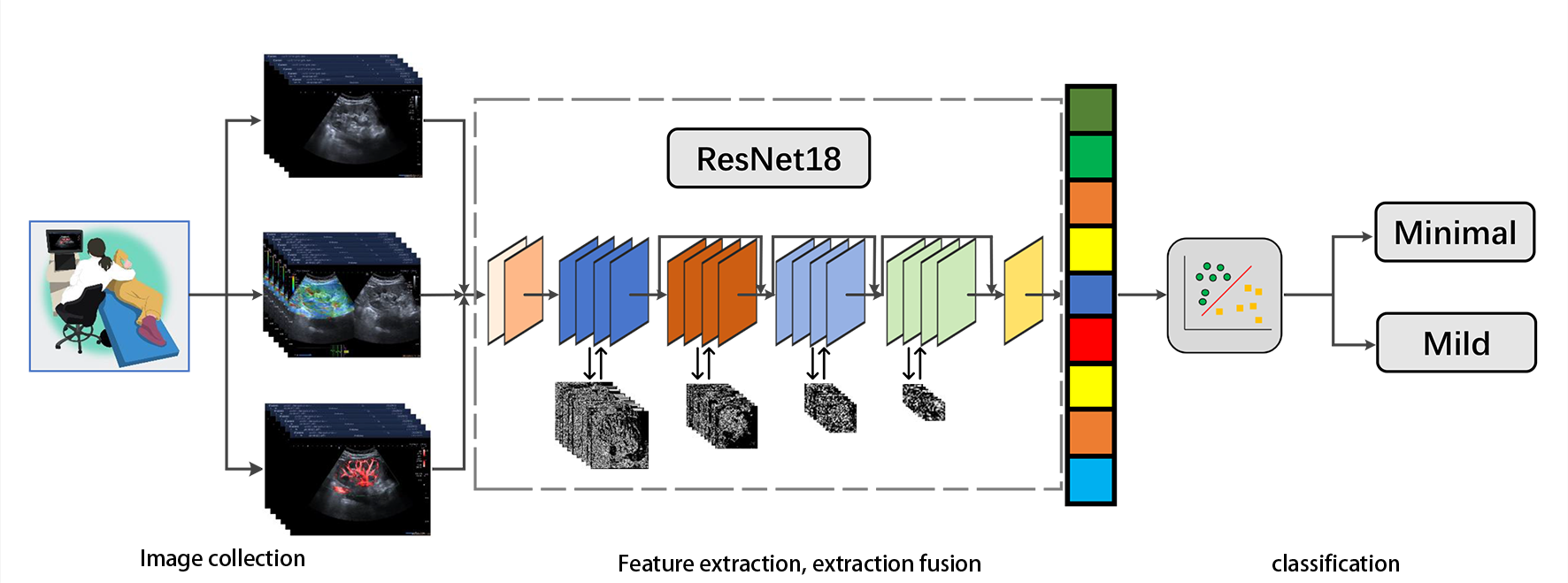

Supplement: fig2_R1.tif [file IRNF_A_2417740_SM4843.tif]

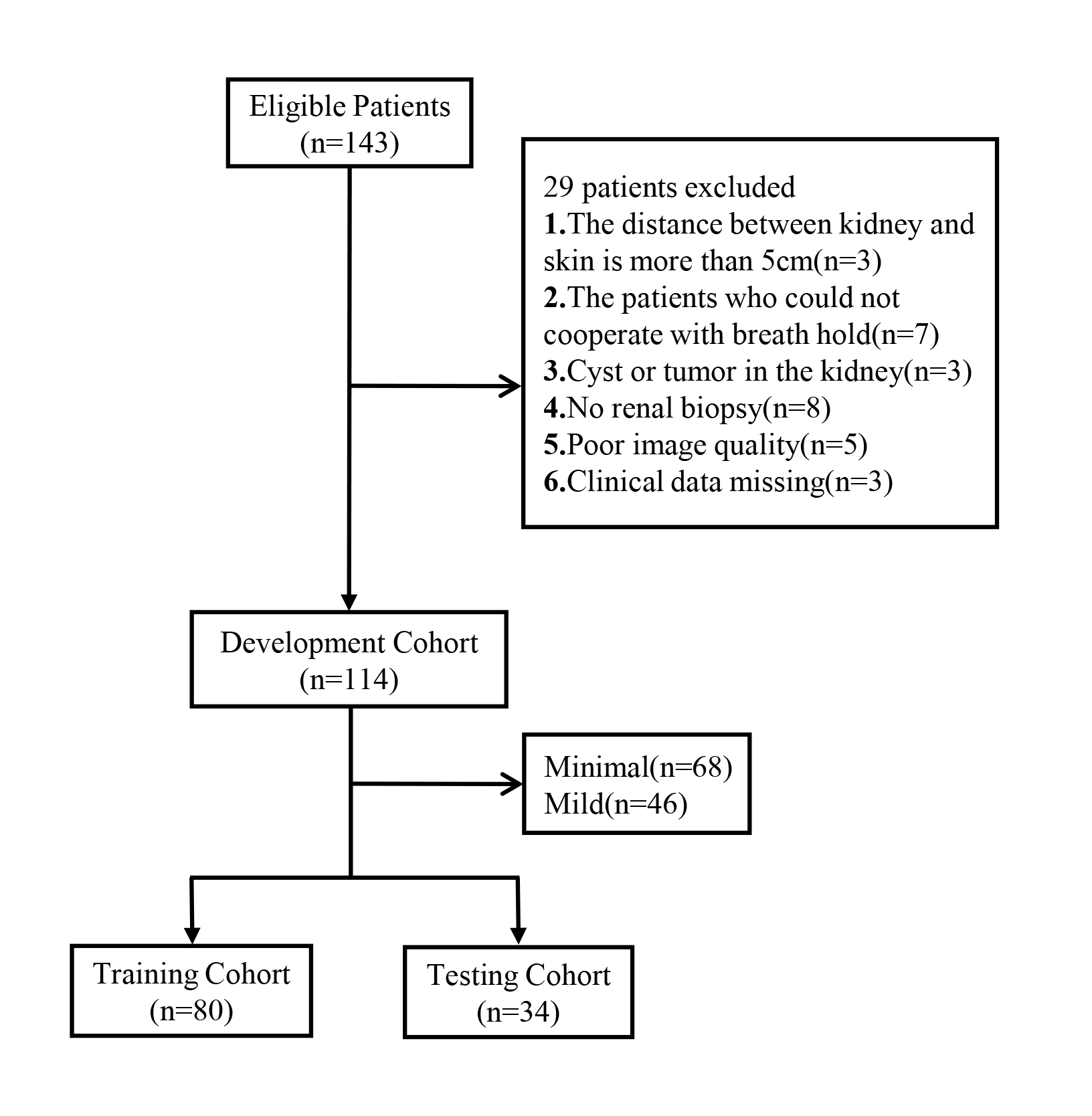

Supplement: fig1_R1.tif [file IRNF_A_2417740_SM4842.tif]
