# Supplementary material for: Multimodal ultrasound deep learning to detect fibrosis in early chronic kidney disease
Source: Ren Fail. 2024 Oct 22;46(2):2417740. doi: 10.1080/0886022X.2024.2417740 (PMC11497579; doi:10.1080/0886022X.2024.2417740)
Supplement: Supplemental file for Review.docx [file IRNF_A_2417740_SM4847.docx]

**Acknowledgment:**

This study has been supported by the Hebin Intelligent Robots Co., LTD.

**Conflict of Interest:**

All the authors declared no competing interests.

**Financial support:**

This study has received funding by the Science and Technology Project of Nanchong City（22JCYJPT0004）.

**Data availability statement:**

The study data may be provided by contacting the corresponding author

**Informed Consent:**

Written informed consent was obtained from all subjects (patients) in this study.

**Ethical Approval:**

Institutional review Board approval was obtained.
