# Supplementary material for: Multimodal ultrasound deep learning to detect fibrosis in early chronic kidney disease
Source: Ren Fail. 2024 Oct 22;46(2):2417740. doi: 10.1080/0886022X.2024.2417740 (PMC11497579; doi:10.1080/0886022X.2024.2417740)
Supplement: Appendix A.docx [file IRNF_A_2417740_SM4844.docx]

***Multimodal ultrasound image collection***

The patient had fasted for >6 h. He performed a breath hold during the procedure. The right kidney was examined by ultrasound. The patient was placed in a left recumbent position. The ultrasound probe was gently positioned in the right abdomen by oblique projection, and a 3.5-MHz probe was used for a kidney ultrasound examination. The probe was placed on the posterior axillary line, and the position and angle of the probe were adjusted to obtain the largest longitudinal gray-scale image of the kidney. Approximately 8−15 pictures were continuously taken for each patient.

The ultrasound scanner was switched to monochrome superb microvascular imaging (SMI) mode. The SMI-specific area of interest frame was placed on the whole kidney. The mechanical index was 1.6, the frame rate was 25–35 frames/s, the dynamic range was 65–75 dB, and the SMI speed was 3.5 cm/s. After obtaining the optimal blood flow section of the kidney, continuous image collection was performed. Approximately 8−15 images were continuously taken for each patient.

For the strain elastography images, the distance between the kidney and skin was <5 cm, and the region of interest was placed on the target kidney. The strain elastography image was generated by gentle, repeated compression with the sensor. We used the quality control indicator at the bottom of the screen to ensure stable image quality, and a pressure bar on the side of the elastic diagram can be seen. The grades from red (soft, maximum elasticity) to green (medium, average strain) to blue (hard, inelastic/strain) give the strain degree in the area of interest. After obtaining a stable strain graph of the kidney, pictures were collected continuously, and 8−15 pictures were collected for each patient.

***Image preprocessing and deep learning model parameters***

In the model training process to expand the amount of training data of the deep learning model, 50% of the images were randomly enhanced using the horizontal and vertical flip functions. The data enhancement process can train more images, effectively simulate the data diversity observed in the real world, and avoid overfitting the model. The image value was adjusted to the range of 0−1 by normalization and standardization before the data were input into the network model, with image resizing to 244×244.

All networks were implemented using the Pytorch deep-learning framework. An **Stochastic Gradient Descent** optimizer was used in the training process. The parameter is a batch size of 256. The learning rate is 0.01 epoch 220, which becomes one-tenth of the previous rate every 60 rounds, with weight-decay of 0.0001 and momentum of 0 9. Owing to the lack of further loss and accuracy improvement, an early stop mechanism was adopted to terminate training.
